# A new cognitive clock matching phenotypic and epigenetic ages

**DOI:** 10.1038/s41398-022-02123-5

**Published:** 2022-09-06

**Authors:** M. I. Krivonosov, E. V. Kondakova, N. A. Bulanov, S. A. Polevaya, C. Franceschi, M. V. Ivanchenko, M. V. Vedunova

**Affiliations:** 1 Institute of Biology and Biomedicine, Department of Neurotechnology, N. I. Lobachevsky State University, Nizhny Novgorod, Russia; 2 Institute of Information Technology, Mathematics and Mechanics, Department of Applied Mathematics, N. I. Lobachevsky State University, Nizhny Novgorod, Russia; 3 Research Center for Trusted Artificial Intelligence, The Ivannikov Institute for System Programming of the Russian Academy of Sciences, Moscow, 109004 Russia; 4 Laboratory of Systems Medicine of Healthy Aging, Institute of Biology and Biomedicine, N. I. Lobachevsky State University, Nizhny Novgorod, Russia; 5 Institute of Biology and Biomedicine, Department of Basic and Medical Genetics, N. I. Lobachevsky State University, Nizhny Novgorod, Russia; 6 Faculty of Computer Science, School of Data Analysis and Artificial Intelligence, HSE University, Moscow, Russia; 7 Faculty of Social Sciences, Department of Psychophysiology, N. I. Lobachevsky State University, Nizhny Novgorod, Russia; 8 Department of Experimental, Diagnostic, and Specialty Medicine (DIMES), University of Bologna, Bologna, Italy

**Keywords:** Human behaviour, Diagnostic markers, Genomics, Epigenetics and behaviour

## Abstract

Cognitive abilities decline with age, constituting a major manifestation of aging. The quantitative biomarkers of this process, as well as the correspondence to different biological clocks, remain largely an open problem. In this paper we employ the following cognitive tests: 1. differentiation of shades (campimetry); 2. evaluation of the arithmetic correctness and 3. detection of reversed letters and identify the most significant age-related cognitive indices. Based on their subsets we construct a machine learning-based Cognitive Clock that predicts chronological age with a mean absolute error of 8.62 years. Remarkably, epigenetic and phenotypic ages are predicted by Cognitive Clock with an even better accuracy. We also demonstrate the presence of correlations between cognitive, phenotypic and epigenetic age accelerations that suggests a deep connection between cognitive performance and aging status of an individual.

## Introduction

In recent decades, there has been a clear trend towards an increase in global life expectancy. A crucial issue in extending lifespan is to make it go with extending health span, characterized by the maintenance of functional capabilities, physical and social activity, and quality of life—in other words, healthy aging. Cognitive status, including memory, thinking, motor reactions and attention, and processing speed, plays a central role in healthy aging. Currently, human cognitive aging remains one of the most interesting and multidisciplinary problems. Studies of age-related changes in the brain at the cellular and systemic levels suggested the concept of “Cognitive aging” [1].

Cognitive decline is responsible for many difficulties even in everyday life, affecting a person’s well-being. The decline in cognitive abilities has been shown to begin during the second decade of life and accelerate as the person ages [2]. However, people of the same chronological age are heterogeneous in the rate of cognitive decline and in the overall level of cognitive impairment. Although age-related neurodegeneration is considered to be a part of the physiological aging process, its acceleration is the reason for the transition from normal functioning to mild cognitive impairment and then to dementia [3]. Thus, changes in the rate of age-related neuronal degradation and changes in cognitive abilities may be responsible for interindividual differences in the manifestation, if any, of dementias, including Alzheimer’s disease (AD), Parkinson’s disease, small vessel disease, and other age-associated pathologies. The ability to define and follow the individual trajectory of age-related cognitive changes could aid to prevent and/or reduce the rate of pathological processes. The capability to easily assess and quantify cognitive aging can also help to identify early factors that influence the rate of age-related neurodegeneration and serve as a fundamental basis for the development of new diagnostic approaches. Over the past 20 years, age-related variations in color perception have attracted considerable attention [4, 5]. The most pronounced age-related effects are associated with the recognition of shades: with age, the sensitivity in the short-wave range of the spectrum decreases more significantly than in the medium- and long-wave ranges. Thus, it becomes more difficult to distinguish colors and perceive color contrasts [6]. Changes in sensorimotor reactions, such as the speed and accuracy of decision-making, are also age-specific. In particular, the study of older adults’ decision-making behavior showed that they use simpler, heuristic strategies [7]. In addition, recent research indicates that response time slowing, which begins as early as age 20, is associated with increased caution in decision-making and slower non-decisional processes, rather than differences in mental speed [8].

Reduced decision-making efficiency has been attributed to cognitive limitations in information processing in older people [9] who have also been shown to be less consistent in their choices [10]. Biologically, these effects have been explained by the loss of neocortical neurons, with the most pronounced atrophic changes observed in the prefrontal cortex. There is also a decrease in the total number of synapses and a decrease in the production of many neurotransmitters [11].

It has been previously demonstrated that age-related cognitive decline, as well as several age-related pathological conditions such as dementia, AD, Parkinson’s, and Huntington’s disease, are associated with epigenetic age acceleration, e.g., [12]. However, robust epigenetic markers of cognitive changes are still lacking, as suggested for example by the lack of correlation of DNAmAge calculated in blood by the Horvath clock with cognitive decline in monozygotic twins [13].

Here we investigate the changes in the cognitive tests performance with age, identify cognitive markers of age and build machine-learning-based models of a cognitive clock. Moreover, we study the correlation of these new cognitive clocks with several epigenetic clocks, and present evidence that the accelerated decline of cognitive performance is linked to accelerated aging. Finally, individuals were clustered in seven groups according to the cognitive tests results. Two of them with considerably better than average campimetry and sensorimotor performance demonstrated statistically significant negative cognitive age acceleration (minus 4.5–6 years), and the other two with the considerably worse performance manifested accelerated cognitive aging (4.5–7 years).

## Materials and methods

The study involved 118 volunteers of both sexes, aged 19–85 years (37 males and 81 females). The exclusion criteria from the study group were acute respiratory infection, oncology, and chronic diseases in the acute stage. Each volunteer signed an informed consent form to participate in the study.

The study employed three cognitive tests: 2 sensorimotor tests (SM1, SM2) and a campimetry test (CM). We chose three different cognitive tests that allow us to characterize the change in psychophysiological reactions of participants from the perspective of age-related decline. Sensorimotor tests measure the sensorimotor response and determine the degree of preservation of brain regions, the resources of spatial and selective attention, as well as the ability to predict and learn [14]. Computer color campimetry measures the color difference function and quantifies subjective psychophysiological responses. This technology makes it possible to characterize the features of the perception of the color and shape of objects and provides information about the level of working memory and attention, as well as the emotional stress of the participants. The selected tests are valid over a wide range of ages and can be applied to participants of different ages.

Tests were performed on Web-platform-ApWay [14]. Supplementary Fig. S1 refers to the stimuli and interface of the corresponding tests on the platform. The detailed description of tests is given in the Supplementary Methods.

### Biological age models

The biological age was estimated based on Phenotypic Age clocks for all samples [15]. The required hematological parameters (WBC, MCV, RDW-CV, LYM (%), albumin, glucose, creatinine, C-reactive protein, alkaline phosphatase) were obtained by complete blood count using a semi-automatic analyzer Abacus Junior 3.0, and a set of biochemical parameters using the StatFax analyzer and diagnostic kits. The group size was limited by the requirement of simultaneous cognitive test and blood samples data acquisition (with the further determination of biological age).

Additionally, four types of epigenetic age were calculated based on DNA methylation analysis of blood samples: DNAmAgeHannum [16], DNAmAge [17], DNAmPhenoAge [15], DNAmGrimAge [18].

The DNA methylation analysis of blood samples was conducted in 47 subjects (20 males and 27 females) out of the 118 subjects. Ages of the whole dataset and subset selected for the DNA methylation analysis were overlapped in the range from 25 to 85 years and equally distributed in that range (Kolmogorov–Smirnov test, *p* value = 0.94). In addition, regardless of sex (males and females), the ages are equally distributed also (female ages: KS-test, *p* value = 0.78, male ages: KS-test, *p* value = 0.9). Similarly, the cognitive test results for the whole dataset and its subset do not exhibit differences between cognitive indices distributions (KS-test, *p* values in the range [0.3; 1.0]).

Extraction of DNA from EDTA whole blood was performed using the phenol-chloroform method. Prior to methylation assay, DNA concentration was determined using the Qubit dsDNA BR Assay kit (Thermo Fisher Scientific). Bisulfite treatment of 250 ng of DNA was performed using an EpiMark bisulfite conversion kit (NEB).

For each sample, DNA methylation profiles were obtained by the Illumina infinium 850 K DNA methylation array. Randomization was employed in all experimental batches. Platform Illumina allows measuring DNA methylation levels from a total number of 866 836 genomic sites, with single-nucleotide resolution. DNA methylation is expressed as β values, ranging from 0 for unmethylated to 1 representing complete methylation for each probe. Raw data were preprocessed as follows. First, probes with a detection *p* value above 0.01 in at least 10% of samples were removed from the analysis. Second, probes with a bead count less than three in at least 5% of samples, were removed from the analysis. Third, all non-CpG probes were excluded from the results [19, 20]. Fourth, SNP-related probes were removed from the analysis [20]. Fifth, multi-hit probes were removed [21]. Sixth, all probes located in chromosome X and Y were filtered out. As a result, 733,923 probes remained for the analysis. All samples have <10% of probes with a detection *p* value above 0.01. Functional normalization of raw methylation data was performed using the minfi R package [22].

### Data analysis

#### Preprocessing procedures

The statistics of cognitive indices that characterize participant responses demonstrate broad distributions, often multimodal, as shown in Supplementary Fig. S4. Therefore, in addition to the mean, we calculate the minimum value, maximum value, standard deviation, median, first and third quartiles of cognitive indices. The transformation of the 16 cognitive index series by computing statistics produced a vector of 64 cognitive quantifiers. Accordingly, each participant was characterized by a vector of cognitive quantifiers, processed by further analysis. The full list of cognitive quantifiers and corresponding indices are presented in Supplementary Table S1 (Cognitive test indices, Cognitive quantifiers).

#### Analysis pipeline

The analysis pipeline consists of three parts: correlation analysis, age prediction by machine learning, and analysis of age accelerations. The analysis against Phenotypic Age [15], DNAmAge [17], DNAmAgeHannum [16], DNAmPhenoAge [15], DNAmGrimAge [18], and chronological age was performed separately. By applying linear analysis, we identified particular age-associated quantifiers and the corresponding cognitive indices. Selected quantifiers enabled to build prediction models for biological and chronological ages. These steps used machine-learning techniques. The quality of age estimation was compared across models. Finally, we perform the correlation analysis of age accelerations.

#### Feature selection

First, we determined cognitive quantifiers that give a statistically significant correlation with all considered ages. The p-value was estimated by testing the null-hypothesis of zero Pearson correlation coefficient ρ using Student’s t-distribution. The produced p-values were corrected using Benjamini–Hochberg procedure [23] and a significance threshold of 0.001 was considered.

#### Machine-learning models

Further, we constructed biological clocks based on the selected cognitive quantifiers. The data was preprocessed by subtracting the mean and normalizing by the standard deviation per individual quantifier. To identify the best model, we examined the following machine-learning models from the scikit-learn package [24]: Elastic Net, Support Vector Machine (with radial basis function as a kernel), Random Forest, Linear Model, k-Nearest Neighbors, Thiel Sen Model.

#### Model selection and evaluation

The quality of predictions was evaluated by the fivefold cross-validation. The data was divided into five equal parts, taking into account the equal age distribution across groups using the Stratified K-Fold algorithm [25]. The stratification algorithm requires multi-class labels, so that the participants were distributed by age into seven bins, ranging from 10 to 90 years old.

The choice of model parameters relied on an optimization search over a multidimensional grid of hyperparameters appropriate for the specific model. In addition, we chose the optimal number of top quantifiers ranked by *p* value for corresponding age characteristics. The objective function was the mean value of the explained variance (EV) computed by fivefold cross-validation.

Evaluation of the quality was based on the EV, mean absolute error (MAE) and median absolute error (MedAE). An optimal model that predicts age based on cognitive quantifiers was defined as Cognitive clocks.

### Biological and cognitive age acceleration analysis

To characterize the deviation of biological and cognitive ages from chronological age we performed correlation analysis. First, we compute age acceleration that describes deviations of the biological age from the expected chronological age for a particular person. Next, we examined correlations between cognitive clock acceleration and other biological clock accelerations. Presence of this correlation indicates that deviations in cognitive clocks are reflected in deviations of the biological clocks.

#### Pattern clustering analysis

**Fig. 1:**
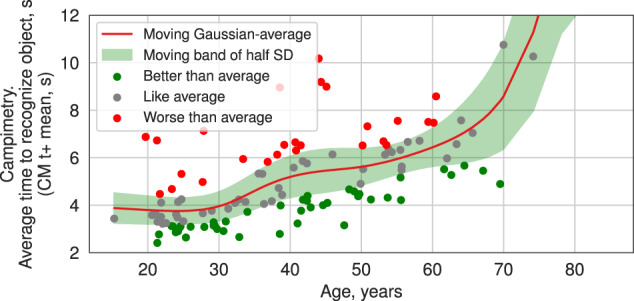
Example of moving Gaussian-weighted average and corresponding moving standard deviation (SD) of cognitive quantifier over age. Subjects were marked as “better performance” (green point)—faster than average by SD, “worse performance” (red point)—slower than average by SD, and “average performance” (gray point).

Decline trajectory of cognitive abilities with age varied widely from one individual to another. We explored this diversity by estimating differences of individual cognitive performance among cohorts of the same age. Subjects were compared to the Gaussian-weighted average of cognitive quantifiers over age relative to current subject (age SD is ±7 years). Deviations of the cognitive quantifier greater than half of Gaussian-weighted SD were considered as “better performance” or “worse performance” depending on the direction (see Fig. 1). All quantifiers associated with time measurement are considered as “better performance” in case of less time spent and as “worse performance” in the opposite case, and less mistakes is better. Both of the campimetry quantifiers are considered as the smaller the better (the number of shade sharpening steps to start distinguishing an object from the background dH+ and the number of shade decay steps until the object merges with the background dH−). In this way, subjects were identified by performance pattern results with three possible outcomes: “better”, “worse”, “normal”. Individuals were grouped by K-means clustering algorithm based on their performance patterns. Finally, the association of groups with cognitive age acceleration were probed by one-sample *T* test by comparing to zero mean (no acceleration).

## Results

### Age-associated cognitive quantifiers

**Fig. 2:**
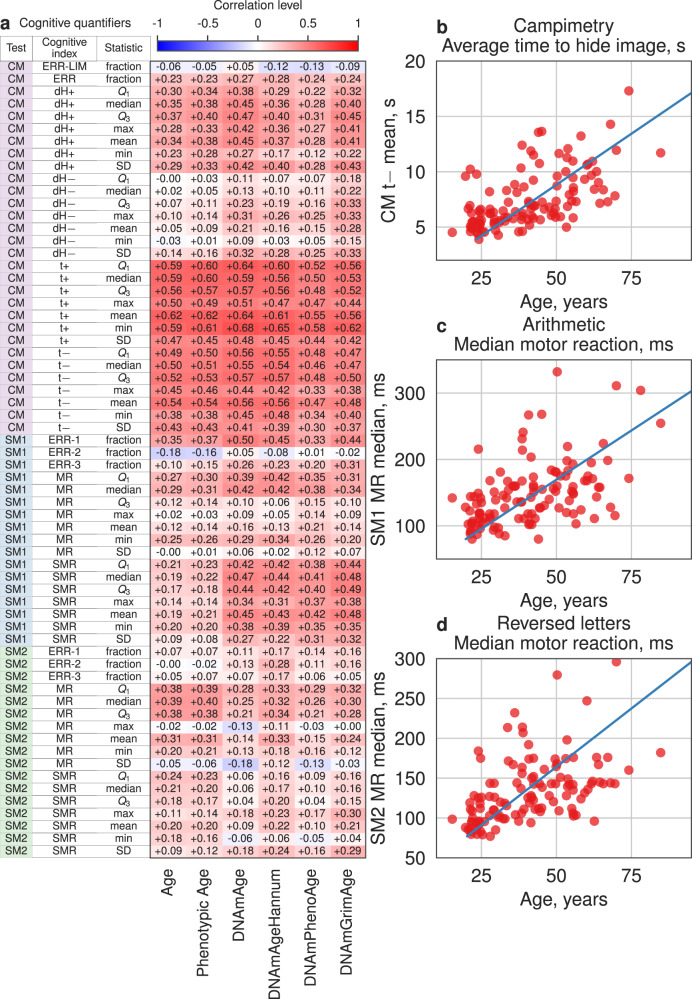
Association of cognitive quantifiers and indices with age. **a** Pearson correlation coefficients resulting from the correlation of cognitive quantifiers and each type of age. **b**–**d** Examples of cognitive quantifiers associated with age.

We started by investigating associations between cognitive quantifiers for individual test series (i.e., mean, standard deviation, median, maximum, minimum values, 1st and 3rd quartiles of cognitive indices, cf. Data Analysis) and chronological age, and found a significant correlation (Benjamini corrected *p* value < 0.001, Pearson correlation) for 21 out of 64 of them (Fig. 2a and Supplementary Table 1). They correspond to the following five cognitive indices: the time of stimulus recognition (CM t+), the time of stimulus hiding (CM t−, see Fig. 2b) and the first recognized shade (CM dH+) in the CM, the fraction of falsely rejected correct arithmetic expressions (SM1 ERR-1, see Fig. 2c) in the arithmetic test, and the motor reaction (SM2 MR, see Fig. 2d) in the reversed letter test. Notably, estimating the correlation between individual cognitive quantifiers and Phenotypic Age [15] produces much similar results to those for the chronological age, manifesting the same cognitive indices (Fig. 2a).

DNA methylation analysis was performed on 54 subjects, i.e., a subset of the whole dataset. The 11 quantifiers show significant correlation (Benjamini corrected *p* value < 0.001, Pearson correlation) with DNAmAge, nine quantifiers with DNAmAgeHannum, five quantifiers with DNAmGrimAge, and only two quantifiers with DNAmPhenoAge, at variance to the above results for the chronological age. The common part of the lists is a CM t+ time of stimulus recognition in the CM, see Fig. 2a and Supplementary Table 1. Although the correlation between specific individual quantifiers and epigenetic ages is greater than with chronological age, the number of quantifiers significantly correlated with epigenetic ages is substantially lower.

### Cognitive clocks

**Fig. 3:**
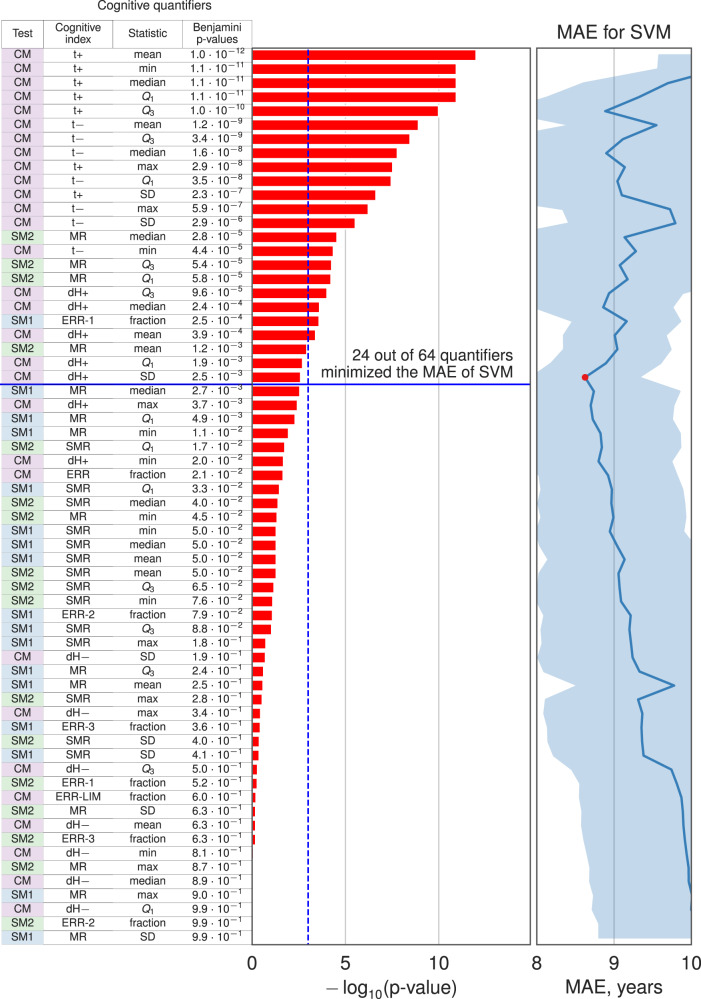
The optimal subset of quantifiers for the Cognitive clock based on the SVM model obtained by minimization of MAE over number of top-ranked quantifiers. Cognitive quantifiers are ordered by their Benjamini *p*-values of Pearson correlation.

**Fig. 4:**
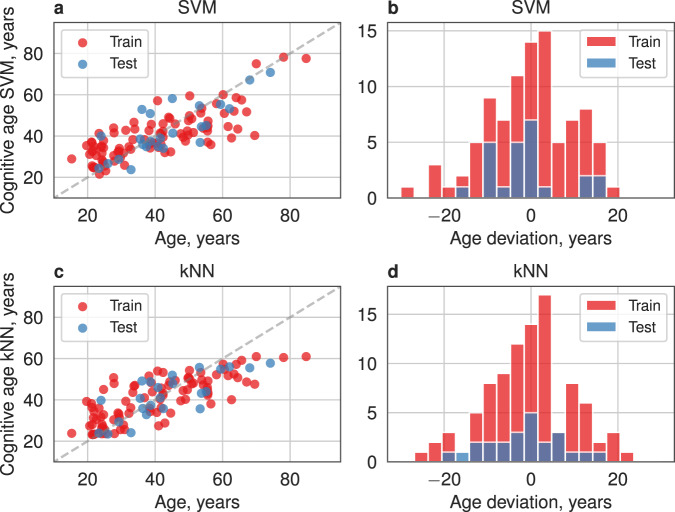
Prediction results of Cognitive age by 2 optimal models: SVM and kNN. Panel rows corresponds to optimal models: SVM (**a**, **b**) and kNN (**c**, **d**). Left column (**a, c**): prediction of chronological age by models for train and test samples; black line corresponds to the diagonal of the first quarter. Right column (**b**, **d**): distribution of age deviations of Cognitive clock. Test set selected as the first 20% of the dataset and train set as the remaining subjects. Such partitions were used only for visualization.

In the next step, we build chronological age predictors based on the top-ranked identified age-associated cognitive quantifiers by employing different machine-learning models (cf. Data Analysis). As a measure of model quality, we used the average EV in the cross-validation. An optimal number of top-ranked cognitive quantifiers was chosen based on maximizing the model quality (See Fig. 3). The average quality of model and its standard deviation in the cross-validation test are reported in Supplementary Table 4. Despite the presence of significant Pearson correlation for quantifiers, the multiple linear regression model shows a lower value of the EV (*R*^2^ = 0.24) in comparison with nonlinear models (SVM with RBF kernel—*R*^2^ = 0.52, MAE = 8.62, kNN—*R*^2^ = 0.51, MAE = 8.66 and Random Forest—*R*^2^ = 0.5, MAE = 8.90), which indicates the presence of nonlinearity in the multidimensional feature space. As a result, out of six examined models, three nonlinear cognitive clocks stood out: SVM, NuSVM, and k-nearest neighbors; their performance is illustrated in Fig. 4. The least MAE of age for those models is 8.62 years and median absolute error of age is 6.25 years. As shown in Fig. 4 the overall quality of both models is similar on training and test datasets, although the SVM model demonstrates a lower error and variance in the prediction of chronological age than kNN. In this way, the optimal model has been shown to predict chronological age based on cognitive test results with good correspondence. In comparison to the other biological clocks, our Cognitive clock model shows comparable performance on the considered dataset. The following enumeration presents MAEs of biological clocks obtained on the studied samples: 3 years for Phenotypic Age, 6.72 years for DNAmAge, 8.2 years for DNAmAgeHannum, 11.89 years for DNAmPhenoAge, 6.18 for DNAmGrimAge.

Based on the minimal mean and variance of MAE we chose the SVM cognitive clock model. The trained model is publicly available at https://github.com/mike-live/cognitive-clock. It incorporates 24 out of 64 cognitive quantifiers that are derivatives of five cognitive indices (See Fig. 3). The CM yields time of stimulus recognition (CM t+), time of stimulus hiding (CM t-) and the first recognized shade (CM dH+). The arithmetic test contribution to cognitive clocks is the fraction of falsely rejected correct arithmetic expressions (SM1 ERR-1). Additionally, the motor reactions (SM2 MR) in the reversed letter test were used as input for machine learning.

Further, we applied the same approach of optimal model construction for age prediction to the rest of biological clocks. The optimal model for predicting Phenotypic Age gives a slightly more accurate result than a model for chronological age prediction (2nd row in Supplementary Fig. S5). Thus, for an optimal NuSVM model, the MAE is 8.25 years, and median absolute error is 5.17 years.

Assessing of epigenetic clocks leads to more accurate results in the terms of prediction based on cognitive quantifiers compared to chronological age and Phenotypic Age. The three epigenetic ages are most closely related to cognitive abilities due to the best-performed MAE: DNAmAge gives 6.25 years, DNAmAgeHannum—6.18 years, and DNAmGrimAge—6.61 years (corresponding median absolute errors in the same order: 5.08 years, 4.01 years, 5.43 years). The prediction of the DNAmAge clock and DNAmAgeHannum clock was constructed on the top-ranked 22 quantifiers (by correlation with corresponding epigenetic age). Those quantifiers are similar for both clocks and include statistics of six indices: time of stimulus recognition (CM t+), time of stimulus hiding (CM t-) and first recognized shade (CM dH+), and also the fraction of falsely rejected correct arithmetic expressions (SM1 ERR-1), the sensorimotor (SM1 SMR) and motor reaction times (SM1 MR) in the arithmetic test. The model that predicts DNAmGrimAge uses only 13 top-ranked features that includes the same indices except the motor reaction time. As illustrated on Supplementary Fig. S5, epigenetic ages (3rd, 4th and 6th rows) outperform other clocks by value of MAE estimated on outcomes of kNN model.

DNAmPhenoAge clock based on PhenotypicAge and DNA methylation, yields to poor quality of prediction by the cognitive quantifiers and high variation of errors in comparison with other clocks (MAE is 9.56, median absolute error is 7.19, see 5th row in Supplementary Fig. S5). The high error variance indicates a lack of robustness in using DNAmPhenoAge as a cognitive age-related measure.

Full cross-validation results of model comparison over each age-characteristics and different approaches to feature selection are presented in Supplementary Table 2.

It is important to note that the data analysis revealed the presence of nonlinearity in the age-related changes of cognitive test statistics of individuals. Despite the presence of separate age-correlated quantifiers, the linear model for predicting the cognitive age of an individual has low robustness due to the high variability in cognitive abilities of the different subjects. In contrast, cross-validation testing of nonlinear models (Support Vector Machine, kNN, Random Forest) shows significantly lower age prediction errors. This indicates the presence of irregular patterns in the changes of cognitive abilities with age.

In addition, the results of the analysis suggest a considerable improvement in the prediction of age processes by biological age regressors, likely a consequence of closer association between cognitive abilities with biological age rather than with chronological age.

### Correlation analysis of age accelerations

**Fig. 5:**
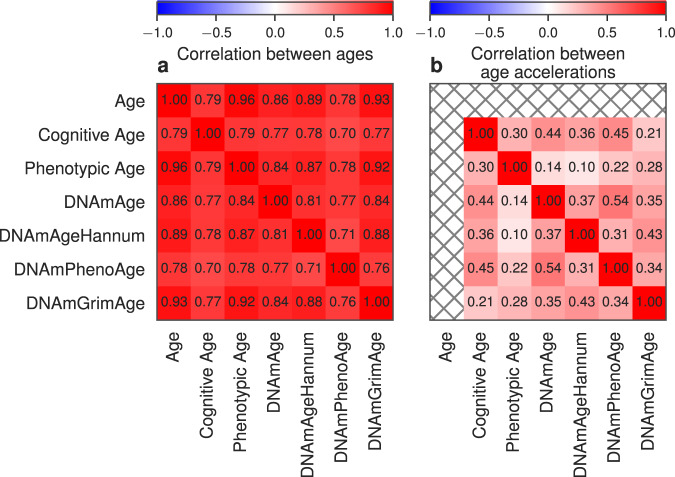
Correlation analysis of ages and age accelerations. Pairwise correlations between ages (**a**) and age accelerations (**b**). The cell of heatmaps shows the Pearson correlation coefficient between different types of ages and age accelerations.

As a next step, we investigated the relationship between epigenetic clocks, phenotypic clock and Cognitive clock. As one would expect, all the clocks showed high correlation (*ρ* > 0.75) with chronological age (Fig. 5a). The correlation between the Cognitive clock and the other clocks is also quite high *ρ* ≈ 0.78 with the exception of DNAmPhenoAge clock (*ρ* = 0.7). On average Cognitive clock shows slightly lower correlations to the other ages than chronological age.

Finally, we performed a correlation analysis between biological age accelerations and Cognitive age accelerations (cf. Data Analysis). Medium correlation levels (*ρ* > 0.3) were found between Phenotypic Age acceleration and Cognitive age acceleration. The correlation level between epigenetic age accelerations and Cognitive age acceleration is slightly higher and corresponds to *ρ* = 0.44 for Horvath clock (DNAmAge), *ρ* = 0.45 for DNAmPhenoAge and *ρ* = 0.36 for DNAmAgeHannum. At variance, DNAmGrimAge accelerations have low correlation level with Cognitive age accelerations. All results of pairwise Pearson correlation values between age accelerations of different types are presented in Fig. 5b. Detailed results of acceleration correlations are given in Supplementary Table 3 and examples of corresponding linear regressions are presented on Supplementary Fig. S6. The presence of medium correlation between age acceleration of Cognitive clocks and the biological clocks suggests that age acceleration in different clocks also reflects changes in individual cognitive abilities with respect to age.

### Clustering of individual behavior patterns

**Fig. 6:**
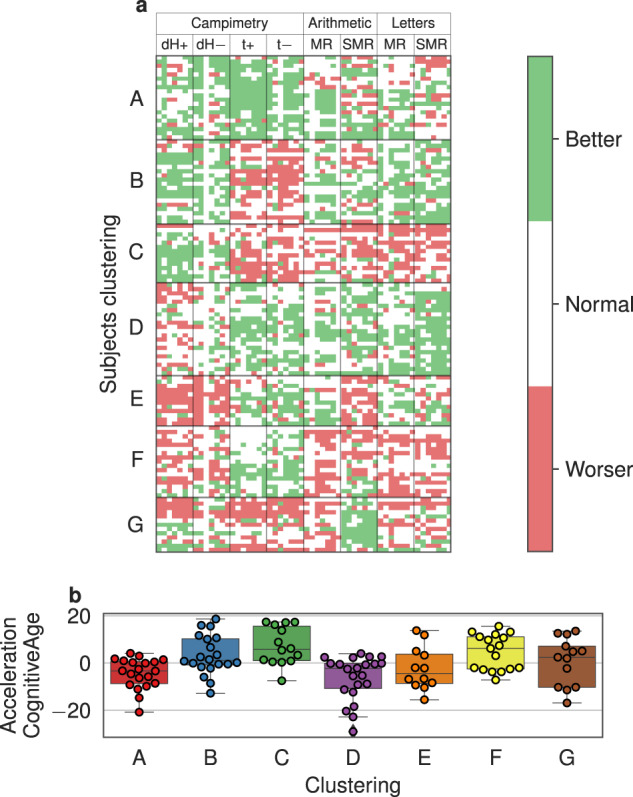
Grouping subjects by similarity of cognitive test results. Rows of the table (**a**) correspond to subjects, columns correspond to cognitive test quantifiers, colors describes the performance of participant relative to others close in age: green—performance is better than average by half SD, red—performance is worse than average by half SD, white—performance like an average. A–G is labels of identified groups. The bottom **b** shows cognitive age acceleration changes in corresponding groups.

First, we defined three possible outcomes of participant performance in the particular cognitive quantifier among the similar age participants: better than average by SD, worse than average by SD, and the rest whose results were average. Next, we found groups of individuals with similar performances. According to the results of cognitive tests, all the subjects were grouped into 7 clusters by their performance relative to similar age participants, denoted by letters from A to G (Fig. 6). It should be noted that in all the groups presented, women prevail as per the whole cohort. Here we analyzed psychophysiological indices related to personal cognitive tests: CM (dH+, dH−, t+, t−) for campimetry and SM1 (MR, SMR), SM2 (MR, SMR) for sensorimotor tests.

Detailed explanations of participant behaviors, their age, and sex in the seven determined groups are given in the Supplementary materials.

Overall, the goal of participants was to adapt their strategy and parameters of their actions to maximize the number of correct answers. Therefore, the results suggest diversity in their approaches.

Finally, we evaluate a new measure of suggested cognitive age by computing the corresponding cognitive age acceleration of participants. The results grouped by clustering analysis results are shown on Fig. 6. According to the results of the acceleration of the cognitive age of the presented groups, it is worth noting a statistically significant accelerated aging for groups C and F (*T* test, *p* value = 0.0051, mean = +7.2 years and *p* value = 0.024, mean = +4.58 years, respectively) and, on the contrary, slow aging for groups A and D (*T* test, *p* value = 0.0062, mean = −4.43 years and *p* value = 0.0054, mean = −6.18 years, respectively). These findings are consistent with the results of clustering after the participants passed cognitive tests. In particular, Group A, which performs excellently on cognitive tests, exhibits delayed aging compared to Groups C and F, which perform less well on all tests and are characterized by accelerated aging. A similar result of the correspondence of the obtained clusters and the acceleration of cognitive age for groups B, C, F, which show less successful results in passing tests compared to group D, which demonstrated delayed aging and good results in all tests. Groups E and G did not show significant changes in age acceleration (*T* test, *p* value = 0.399 and 0.822, respectively).

However, no statistically significant relationships were found between the groups in terms of chronological age (Supplementary Fig. S7), and different types of biological age. Biological age accelerations did not show statistically significant differences too (*T* test, *p* value > 0.05).

## Discussion

Currently, there is no doubt that the aging processes inter alia affect human cognitive abilities. Even with physiological aging, there is a decrease in reaction speed, while intellectual abilities, such as conceptual thinking ability, generalization, memory reconsolidation, and spatial orientation, can remain at a high level. Biologically, physiological aging is accompanied by age-related neurodegeneration (a decrease in the number of neurons in the brain), impaired energy metabolism, and degradation of astrocytes, which can be considered a factor in reducing the number and quality of the pool of synaptic contacts. The causes of systemic changes include atherosclerosis, the persistence of senescent cells in neuronal circuits, a body–brain trophic interactions, and lifespan gut dysbiosis [26]. However, the rate of age-related brain changes varies across individuals and does not correlate with the general state of the body. A fundamental question remains: given the huge variability of individual brain developmental tracks, are there cognitive indicators correlating with the biological and chronological age of an individual? We have used two methods for determining biological age, the correlation of the indicators of cognitive tests and biological age. The first method is determining the deviation of biological age from chronological age by a set of biochemical and hematological markers [15]. The second method is determining biological age by the level of DNA methylation, i.e. epigenetic age. It is known that the genomic DNA methylation landscape changes with age [16, 27, 28] and DNA methylation (compared for example to telomere length measurement) allows determining the biological age of an individual with an accuracy of ±5 years [29]. The maximum correlation coefficients between epigenetic age and cognitive skills were obtained for some indicators of computer campimetry and sensorimotor reactivity tests: visual stimulus recognition time (CM t+) and total sensorimotor reaction time (SMR), respectively. Contrary to our expectation, the indicators related to the psycho-emotional sphere and color perception showed the highest correlation between chronological, biological, and epigenetic age. At the same time, the indicators that directly characterize logical thinking and the speed of the sensorimotor reaction were not so significant in our studies. This fact is of fundamental importance since our study aimed to identify indicators of age-related physiological changes.

Based on this, age-related changes in color perception can be explained by an increase in the proportion of neurons with somatic mutations caused by epigenetic drift in the corresponding neural networks [30, 31]. With aging, structural and functional losses in the organization of the receptor apparatus of neurons, channel proteins, enzymes, mediator peptides, etc., are inevitable. Obviously, as the biological age of an individual increases, these disorders have more negative effects on primary cognitive functions: sensory, perceptual, mnestic ones.

The direct correlation of epigenetic age with the time of the sensorimotor reaction may indicate a natural decrease in the speed of information processing in the nervous system with age, and the revealed correlation between the methylation level and the latency period of pattern recognition in the computer CM is likely caused by a decrease in the efficiency of signal extraction from noise.

The influence of age-related processes on the cognitive status of a person makes it possible to construct a cognitive clock on the basis of chronological and different types of biological age. The most accurate predictions of epigenetic age using the cognitive status of a person were achieved for DNAmAge [17] and DNAmAgeHannum [16], constructed by the chronological age fitting. On the other hand, biological clock models that take into account the results of the blood biochemistry, smoking, and death risk markers show less accurate prediction results from the perspective of cognitive quantifiers.

The estimated cognitive age of subjects usually does not precisely coincide with the chronological age. Such arising deviations can be partly explained by deviations of epigenetic age that occur due to alterations in the methylation profile. The results suggest that cognitive age acceleration is mostly associated with the epigenetic age acceleration estimated by Horvath Clock and less associated with Phenotypic Age acceleration derived from blood chemistry.

This finding in turn reinforces the hypothesis that changes in the human cognitive system are not only linked with the processes that determine blood biochemistry but also reflected in the age-associated regions of the DNA methylation profile.

Remarkably, the clustering analysis reveals seven groups in the corresponding performances relative to the similar age individuals. Comparing the selected clusters with each other, it is interesting to note the difference in the results between groups D and G, in which the difference in age reaches 10 years. According to literature data, it is known that in middle age women have better memory and information processing speed than men. This female advantage decreases with age (women show faster cognitive decline) and increases in later age cohorts [32]. It is important to take into account that a large number of factors can influence the decline in cognitive functions with respect to age. In particular, some studies report an association between cardiovascular diseases and their risk factors with cognitive decline in middle age [33].

In addition, the intellectual characteristics of a person, including the level of intelligence and cognitive reserve (the level of education and the complexity of the profession), affect the accuracy of the task [34], as well as physical activity, which correlates with a decrease in cognitive functions in age aspect [35].

Finally, a recent multi-omics study of one of us in young adults shows that in each individual the different organs and systems of the body age at different rates [36], concordantly with the earlier findings for mice [37].

Although the constructed cognitive clock has already proved a robust and powerful tool, the study is not free from limitations. First, the size of the cohort should be substantially increased, improving the model performance and decreasing its error. Another potential confounding factor that should be rectified in the future is the prevalence of females over males in the collected dataset, although a statistically significant difference between the male and female test results is not currently found.

We expect that the proposed method of cognitive age estimation, beside revealing markers of accelerated aging, has a potential for assessing age-related acceleration associated with the early manifestation of neurodegenerative diseases.

### Ethic statements

The studies involving human participants were reviewed and approved by the Local ethics committee of Lobachevsky State University, Nizhny Novgorod, Russia (protocol no.1, 2020 year).

## Supplementary information

Supplementary Materials

Supplementary Figure S1

Supplementary Figure S2

Supplementary Figure S3

Supplementary Figure S4

Supplementary Figure S5

Supplementary Figure S6

Supplementary Figure S7

Supplementary Table S1

Supplementary Table S2

Supplementary Table S3

## Data Availability

The data are available from the authors upon request and subject to the approval of the Local Ethical Committee of Lobachevsky University.

## Code availability

The code to reproduce the results of computing Cognitive age by the SVM model is available in the Github repository by the link https://github.com/mike-live/cognitive-clock.

## References

[1] Blazer DG (2017). CognitIve aging: what we fear and what we know. Perspect Biol Med.

[2] Singh-Manoux A, Czernichow S, Elbaz A, Dugravot A, Sabia S, Hagger-Johnson G (2012). Obesity phenotypes in midlife and cognition in early old age. Neurology.

[3] Levine ME, Harrati A, Crimmins EM (2018). Predictors and implications of accelerated cognitive aging. Biodemography Soc Biol.

[4] Karwatsky P, Overbury O, Faubert J (2004). Red-green chromatic mechanisms in normal aging and glaucomatous observers. Invest Ophthalmol Vis Sci.

[5] Wagner H-J, Kröger RHH (2005). Adaptive plasticity during the development of colour vision. Prog Retin Eye Res.

[6] Nguyen-Tri D, Overbury O, Faubert J (2003). The role of lenticular senescence in age-related color vision changes. Invest Ophthalmol Vis Sci.

[7] Queen TL, Hess TM, Ennis GE, Dowd K, Grühn D (2012). Information search and decision making: effects of age and complexity on strategy use. Psychol Aging.

[8] von Krause M, Radev ST, Voss A. Mental speed is high until age 60 as revealed by analysis of over a million participants. Nat Hum Behav. 2022;6:700–8. 10.1038/s41562-021-01282-7 35177809

[9] Frey R, Mata R, Hertwig R (2015). The role of cognitive abilities in decisions from experience: age differences emerge as a function of choice set size. Cognition.

[10] Finucane ML, Mertz CK, Slovic P, Schmidt ES (2005). Task complexity and older adults’ decision-making competence. Psychol Aging.

[11] Fjell AM, Walhovd KB (2010). Structural brain changes in aging: courses, causes and cognitive consequences. Rev Neurosci.

[12] Horvath S, Raj K (2018). DNA methylation-based biomarkers and the epigenetic clock theory of ageing. Nat Rev Genet.

[13] Starnawska A, Tan Q, McGue M, Mors O, Børglum AD, Christensen K (2017). Epigenome-wide association study of cognitive functioning in middle-aged monozygotic twins. Front Aging Neurosci.

[14] Polevaya SA, Eremin EV, Bulanov NA, Bakhchina AV, Kovalchuk AV, Parin SB (2019). Event-related telemetry of heart rate for personalized remote monitoring of cognitive functions and stress under conditions of everyday activity. Sovrem Tehnol V Med.

[15] Levine ME, Lu AT, Quach A, Chen BH, Assimes TL, Bandinelli S (2018). An epigenetic biomarker of aging for lifespan and healthspan. Aging.

[16] Hannum G, Guinney J, Zhao L, Zhang L, Hughes G, Sadda S (2013). Genome-wide methylation profiles reveal quantitative views of human aging rates. Mol Cell.

[17] Horvath S (2013). DNA methylation age of human tissues and cell types. Genome Biol.

[18] Lu AT, Quach A, Wilson JG, Reiner AP, Aviv A, Raj K (2019). DNA methylation GrimAge strongly predicts lifespan and healthspan. Aging.

[19] McCartney DL, Walker RM, Morris SW, McIntosh AM, Porteous DJ, Evans KL (2016). Identification of polymorphic and off-target probe binding sites on the Illumina Infinium MethylationEPIC BeadChip. Genomics Data.

[20] Zhou W, Laird PW, Shen H (2017). Comprehensive characterization, annotation and innovative use of Infinium DNA methylation BeadChip probes. Nucleic Acids Res.

[21] Nordlund J, Bäcklin CL, Wahlberg P, Busche S, Berglund EC, Eloranta M-L (2013). Genome-wide signatures of differential DNA methylation in pediatric acute lymphoblastic leukemia. Genome Biol.

[22] Aryee MJ, Jaffe AE, Corrada-Bravo H, Ladd-Acosta C, Feinberg AP, Hansen KD (2014). Minfi: a flexible and comprehensive Bioconductor package for the analysis of Infinium DNA methylation microarrays. Bioinforma Oxf Engl.

[23] Benjamini Y, Hochberg Y (1995). Controlling the false discovery rate: a practical and powerful approach to multiple testing. J R Stat Soc Ser B Methodol.

[24] Pedregosa F, Varoquaux G, Gramfort A, Michel V, Thirion B, Grisel O, et al. Scikit-learn: Machine Learning in Python. J Mach Learn Res. 2011;6.

[25] James G, Witten D, Hastie T, Tibshirani R. An Introduction to Statistical Learning. Vol. 103, Springer Texts in Statistics (New York, Springer; 2013).

[26] Castillo X, Castro-Obregón S, Gutiérrez-Becker B, Gutiérrez-Ospina G, Karalis N, Khalil A, et al. Re-thinking the etiological framework of neurodegeneration. Front Neurosci. 2019;13:728. 10.3389/fnins.2019.00728 PMC6667555 31396030

[27] Johansson A, Enroth S, Gyllensten U (2013). Continuous aging of the human DNA methylome throughout the human lifespan. PloS One.

[28] Florath I, Butterbach K, Müller H, Bewerunge-Hudler M, Brenner H (2014). Cross-sectional and longitudinal changes in DNA methylation with age: an epigenome-wide analysis revealing over 60 novel age-associated CpG sites. Hum Mol Genet.

[29] Weidner CI, Lin Q, Koch CM, Eisele L, Beier F, Ziegler P (2014). Aging of blood can be tracked by DNA methylation changes at just three CpG sites. Genome Biol.

[30] Teschendorff AE, West J, Beck S (2013). Age-associated epigenetic drift: implications, and a case of epigenetic thrift?. Hum Mol Genet.

[31] West J, Widschwendter M, Teschendorff AE (2013). Distinctive topology of age-associated epigenetic drift in the human interactome. Proc Natl Acad Sci.

[32] Nooyens ACJ, Wijnhoven HAH, Schaap LS, Sialino LD, Kok AAL, Visser M, et al. Sex differences in cognitive functioning with aging in the Netherlands. Gerontology. 2022;1–11. 10.1159/000520318 PMC9501735 34983049

[33] Huo N, Vemuri P, Graff-Radford J, Syrjanen J, Machulda M, Knopman DS (2022). Sex differences in the association between midlife cardiovascular conditions or risk factors with midlife cognitive decline. Neurology.

[34] Turcotte V, Potvin O, Dadar M, Hudon C, Duchesne S (2022). Initiative for the ADN. Birth cohorts and cognitive reserve influence cognitive performances in older adults. J Alzheimers Dis.

[35] Krell-Roesch J, Syrjanen JA, Bezold J, Trautwein S, Barisch-Fritz B, Boes K, et al. Physical activity and trajectory of cognitive change in older persons: mayo clinic study of aging. J Alzheimers Dis. 2021;79:377–88. 10.3233/JAD-200959 PMC7839815 33216032

[36] Nie C, Li Y, Li R, Yan Y, Zhang D, Li T (2022). Distinct biological ages of organs and systems identified from a multi-omics study. Cell Rep.

[37] Schaum N, Lehallier B, Hahn O, Pálovics R, Hosseinzadeh S, Lee SE (2020). Ageing hallmarks exhibit organ-specific temporal signatures. Nature.

