# Correction to: A new cognitive clock matching phenotypic and epigenetic ages

**DOI:** 10.1038/s41398-022-02219-y

**Published:** 2022-10-19

**Authors:** M. I. Krivonosov, E. V. Kondakova, N. A. Bulanov, S. A. Polevaya, C. Franceschi, M. V. Ivanchenko, M. V. Vedunova

**Affiliations:** 1 Institute of Biology and Biomedicine, Department of Neurotechnology, N. I. Lobachevsky State University, Nizhny Novgorod, Russia; 2 Institute of Information Technology, Mathematics and Mechanics, Department of Applied Mathematics, N. I. Lobachevsky State University, Nizhny Novgorod, Russia; 3 Research Center for Trusted Artificial Intelligence, The Ivannikov Institute for System Programming of the Russian Academy of Sciences, Moscow, 109004 Russia; 4 Institute of Biology and Biomedicine, Department of Basic and Medical Genetics, N. I. Lobachevsky State University, Nizhny Novgorod, Russia; 5 Faculty of Computer Science, School of Data Analysis and Artificial Intelligence, HSE University, Moscow, Russia; 6 Faculty of Social Sciences, Department of Psychophysiology, N. I. Lobachevsky State University, Nizhny Novgorod, Russia; 7 Department of Experimental, Diagnostic, and Specialty Medicine (DIMES), University of Bologna, Bologna, Italy

**Keywords:** Human behaviour, Diagnostic markers, Genomics, Epigenetics and behaviour

Correction to: *Translational Psychiatry* 10.1038/s41398-022-02123-5, published online 06 September 2022

The original version of this article contained an error in affiliation 3 of the author Mikhail Krivonosov. The correct affiliation is: Research Center for Trusted Artificial Intelligence, The Ivannikov Institute for System Programming of the Russian Academy of Sciences, Moscow, Russia 109004. In addition, there was an error in the acknowledgements. It should read: “We acknowledge non-financial support by the project of the Ministry of Education and Science of the Russian Federation Agreement No. 075-15-2021-639. The part on machine-learning and clustering analysis acknowledges a grant for research centers in the field of artificial intelligence, provided by the Analytical Center for the Government of the Russian Federation in accordance with the subsidy agreement (agreement identifier 000000D730321P5Q0002) and the agreement with the Ivannikov Institute for System Programming of the Russian Academy of Sciences dated November 2, 2021 No. 70-2021-00142.”. The original article has been corrected.

