# Supplementary material for: A new cognitive clock matching phenotypic and epigenetic ages
Source: Transl Psychiatry. 2022 Sep 6;12:364. doi: 10.1038/s41398-022-02123-5 (PMC9444998; doi:10.1038/s41398-022-02123-5)
Supplement: Supplementary file 1 — Supplementary Materials [file 41398_2022_2123_MOESM1_ESM.doc]

Supplementary materials:

Tables S1-S5

**Supplementary Table S1.**

Sheets:

- Cognitive test indices – list of estimated measures for each participant action
- Cognitive quantifiers – list of sample statistics computed on test indices.
- Summary – lists of significantly correlated quantifiers with particular type of age.
- Short summary– lists of indices corresponding to significantly correlated quantifiers with particular type of age.
- Age, DNAmAge, DNAmAgeHannum, DNAmGrimAge, DNAmPhenoAge, PhenoAge – full lists of quantifier correlations with particular age.

**Supplementary Table S2.**

Sheets:

- Summary best model – quality of optimally selected machine learning models on different types of age.
- Summary MedAE – median absolute deviation values computed for different approaches for quantifier selection, machine learning models and types of age.

**Supplementary Table S3.**

Sheets:

- Summary accelerations (rho) – correlation between accelerations for optimal models in the class and particular type of age. Accelerations computed over chronological age.

| **Model** | **Explained variance** | **Mean**  **absolute error,**  **years** | **Median  absolute error,**  **years** |
| --- | --- | --- | --- |
| **ElasticNet** | 0.38 ± 0.16 | 10.69 ± 1.31 | 9.66 ± 1.09 |
| **Linear Regression** | 0.24 ± 0.12 | 10.99 ± 1.11 | 8.24 ± 1.51 |
| **SVM** | 0.52 ± 0.09 | **8.62 ± 0.70** | 6.25 ± 1.67 |
| **NuSVM** | 0.53 ± 0.14 | 8.84 ± 0.92 | 6.39 ± 1.15 |
| **Random Forest** | 0.50 ± 0.09 | 8.90 ± 0.86 | 6.60 ± 1.22 |
| **k-nearest neighbors** | 0.51 ± 0.09 | **8.66 ± 1.12** | 6.39 ± 1.12 |
| **Theil-Sen Regression** | 0.15 ± 0.22 | 11.43 ± 1.16 | 7.85 ± 1.77 |

**Supplementary Table S4.** The quality of chronological age estimation by the optimal in a class machine learning model. The best performance is demonstrated by the **k-nearest neighbors** algorithm and **SVM** algorithm**.** The **SVM** algorithm leads to lower variance of mean absolute error in the cross-validation procedure comparing to **kNN**.

| **Group** | **Average age** | **Strategy** |
| --- | --- | --- |
| **A** | 39.2 | CM: faster and better color discrimination  SM: more time – higher accuracy |
| **B** | 40.0 | CM: more time – better color discrimination  SM: faster and higher accuracy |
| **C** | 44.5 | CM: more time – better color discrimination  SM: slower and lower accuracy |
| **D** | 37.8 | CM: less time – worse color discrimination level  SM: faster and higher accuracy |
| **E** | 46.0 | CM: less time – worse color discrimination level  SM: faster click – slower decision-making and lower accuracy |
| **F** | 37.9 | CM: less time – worse color discrimination level  SM: slower and lower accuracy |
| **G** | 47.4 | CM: more time and worse color discrimination level  SM: slower and lower accuracy  Except faster decision-making in arithmetic |

**Supplementary Table S5.** Descriptive statistics and the corresponding strategy of identified groups of participant performances relative to their age.

Figures S1-S7

**Supplementary Figure S1.** Examples of visual stimuli used in cognitive tests and test obtained results.

**Supplementary Figure S2.** Scheme of sensorimotor tests: arithmetic expressions (and reversed letters). The purple arithmetic expressions are an example of correct and incorrect stimuli that corresponds to green and red bars on the timeline respectively. The clicks of participant shown as blue bars with start and stop click times. Right choice is a click on correct stimuli and skip incorrect stimuli. Missing of target stimulus corresponds to a type 1 error (ERR-1). Double-click (two blue bars on a green column) – a type 2 error (ERR-2). Click on incorrect stimulus (red bar with a vertical blue stick) – a type 3 error (ERR-3).

**Supplementary Figure S3.** Scheme of campimetry test. A. Stages of changing visual context during performing a campimetry test by participant (direct task). Incorrectly identified target stimulus led to a detection error (ERR-1). B. Stages of changing visual context during performing a campimetry test by participant (reverse task). Tries to decrease hue for object that already equals to background evaluated as limit error (ERR-LIM). (Shades of the example object are stressed to show the idea of test independently of reader screen).

**Supplementary Figure S4.** Typical examples of sensorimotor reaction time distributions (SMR) in the arithmetic test (SM2).

**Supplementary Figure S5.** The x-axis corresponds to the model error in years (the bars shows median absolute errors averaged over 5-folds in the cross-validation procedure and its standard deviations). The rows represent the result of the particular age estimation by the corresponding optimal model. Optimal models were selected by minimization of mean absolute error over number of top age-related quantifiers.

**Supplementary Figure S6.** The regression and Pearson correlation between Cognitive age acceleration and different biological age accelerations.

**Supplementary Figure S7.** Distribution of ages within identified groups.

Supplementary Methods

Description of cognitive tests

Sensorimotor test

Sensorimotor tests involved (SM1) arithmetic calculations and (SM2) identification of reversed letters. The participants were required to press the left button of a computer mouse upon presentation of a target stimulus (correct arithmetic or spelling), while the erroneous stimulus was to be ignored. In total, 15 arithmetic expressions (SM1) and 50 stimuli (letters of the native alphabet) (SM2) were presented. Stimuli appeared and disappeared at a certain pre-programmed rate (4 seconds for SM1 and 2 seconds for SM2). Duration of stimuli showing is 2 seconds in the arithmetic test, and 300 ms in the reversed letter test. The scheme of the sensorimotor test is shown on the Supplementary Fig. 2. As a cognitive test result were evaluated the following times and errors. First, the sensorimotor response (SMR) is the time from the moment the stimulus is detected to the pressing of the button finishes (click); the motor response (MR) is the time the computer mouse button is held down. Multiple indicators, including errors in signal detection and identification, characterized the participants’ responses to stimuli (Supplementary Table 1, Cognitive test indices).

Campimetry test

Computer campimetry is a technology that allows to measure differential thresholds using color model coordinates in a virtual computer environment. The result of testing in campimetry is the threshold values of shades for each of the presented stimuli. Differential thresholds are minimal distinctions between two values of a parameter that cause difference in feelings. Color difference function describes the distribution of differential thresholds on a scale of shades. Multiple color models can be used for defining color shades. The set of stimuli is formed inside the color virtual HSL (stands for Hue, Saturation and Lightness) model, which uses hue, saturation and lightness as the main coordinates. Each stimulus consists of a color background (background stimulus) and a color pattern (target stimulus) within the background.

The method consists of two phases. At first, the subject is presented with a stimulus against a background of the same (stimulus’) color. At the initial presentation of each stimulus to the participant, the values of the shades of the background and the target stimulus are equal. Then, the subject changes the hue of the stimulus by pressing the left mouse button until they can see and indicate what object was “hidden” in the given background. The task of the participant is to determine the shape of the target stimulus in the background, increasing the value of its hue in relation to the hue of the background. Next, they solve the reverse task – as the object changes its hue back to the background color, they need to capture the moment when the object becomes no longer noticeable. The scheme of campimetry test is presented on Supplementary Fig. 3.

Responses of the participant to the stimuli were characterized by multiple indices:

1. dH+ – Difference between stimulus hue and background hue at the time of stimulus detection;
2. dH- – Difference between stimulus hue and background hue for reverse task;
3. t+ – Time between the moment of presentation of the color window and the moment of stimulus detection;
4. t- – Time between the start of the reverse task and the moment the stimulus is lost;
5. ERR – Detection error. The participant chosen wrong object;
6. ERR-LIM – Limit error during inverse task. The perception of participant saves image, but object already disappeared in the background.

Supplementary description of clustered groups

The summarized group description is presented in the Supplementary Table 5. Here we consider the detailed description of groups:

• Group A – men and women, average age 39.2 years. Successful performance of all the presented tests compared to participants of the same age is noted, with the exception of the sensorimotor reaction (time for stimulus recognition and decision-making) in the test for distinguishing letters of the alphabet. This cluster of participants spends more time making decisions in the context of this task, however, at the same time, this group showed the least number of errors of type 1 (ERR-1) – omission / lack of response to the right stimulus and type 3 (ERR-3) – reaction to wrong stimulus. Thus, this group presumably chooses the strategy "more time – more precisely the result". The results obtained indicate a high concentration of attention and a good working memory.

• Group B – men and women, average age 40.0 years. This cluster is characterized by the successful completion of all tasks in sensorimotor tests. Participants spend little time getting an accurate result (the average number of all types of errors is less than or equal compared to other groups). A feature is the performance of the campimetry test: this cluster of participants spends more time to recognize the target stimulus, while using the minimum number of clicks. Thus, this group, in the context of performing the task of distinguishing objects, chooses the strategy “more time – less clicks”. The results of sensorimotor tests indicate a high resource of attention and good learning ability.

• Group C is a small group of men and women, average age 44.5 years. Less successful performance of all the presented tests is noted in comparison with participants of similar age. This cluster spends a lot of time solving all the presented tasks, both motor and sensorimotor, and shows a large number of all types of errors in sensorimotor tests. At the same time, in the campimetry test, the participants in this cluster had a lower color discrimination threshold for objects, but they spend more time on this task. Thus, this group is characterized by a low signal transmission rate in neural networks, which may be associated with age-related changes.

• Group D – men and women, average age 37.8 years. This cluster successfully performs all types of sensorimotor tests, spends little time on decision-making, while showing high accuracy (low level of errors of the ERR-1 and ERR-3 types – skipping the right stimuli and reacting to the wrong stimuli). However, this cluster is less successful in the task of color discrimination and demonstrates a high level of errors ERR-LIM – exceeding the maximum possible number of clicks for the perception of objects when solving the inverse problems in the campimetry test. Thus, this group, in the context of performing the task of distinguishing objects, chooses the strategy “less time – more clicks”. Perhaps there is a “stuck” effect in which a person sees an object when it actually disappears from the background, which may be a feature of color perception.

• Group E is a small group of men and women, average age 46.0 years. This cluster of participants shows features in performing tasks of both sensorimotor tests and color discrimination in the campimetry test. Participants in this group spend a lot of time recognizing the stimulus and making decisions on the arithmetic test, while making a large number of errors, demonstrating a reaction to the wrong stimulus. In the campimetry test, they choose the strategy of applying more clicks with the least amount of time spent, which is reflected in the number of ERR-LIM errors.

• Group F – men and women, average age 37.9 years. This cluster is characterized by unsuccessful performance of all types of tasks in comparison with participants of similar age. People in this group spend more time on all tasks, both motor and sensorimotor. When performing a campimetry test, they spend less time, but at the same time, they show a high color discrimination threshold and a large number of errors, including errors in the detection of figures (ERR-1). These results indicate a reduced concentration of attention and a decrease in working memory.

• Group G is a small group of men and women, average age 47.4 years. This cluster is characterized by unsuccessful performance of all types of tasks in comparison with participants of the same age. The exception is the sensorimotor reaction in the test for arithmetic calculations, where the participants in this cluster show good results. However, when performing all cognitive tests, they make a large number of errors of various kinds (including the maximum number of errors among other clusters in the campimetry test). These results may indicate a decrease in concentration, working memory and distortion of the color discrimination function, which may be associated with age-related changes.
