# Supplementary figures and images for: A new cognitive clock matching phenotypic and epigenetic ages
Source: Transl Psychiatry. 2022 Sep 6;12:364. doi: 10.1038/s41398-022-02123-5 (PMC9444998; doi:10.1038/s41398-022-02123-5)

1+2=3 >

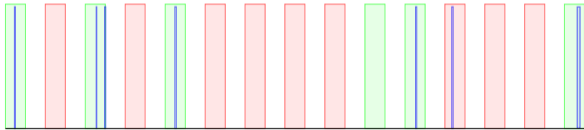

Sensomotor test results

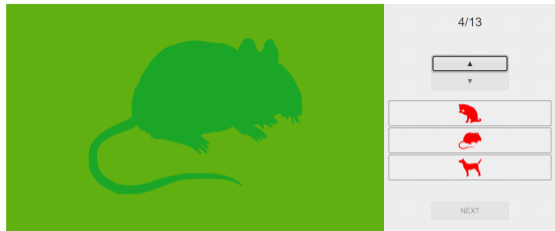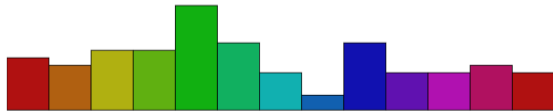

Campimetry test results

Supplement: Supplementary file 2 — Supplementary Figure S1 [file 41398_2022_2123_MOESM2_ESM.pdf]

**a**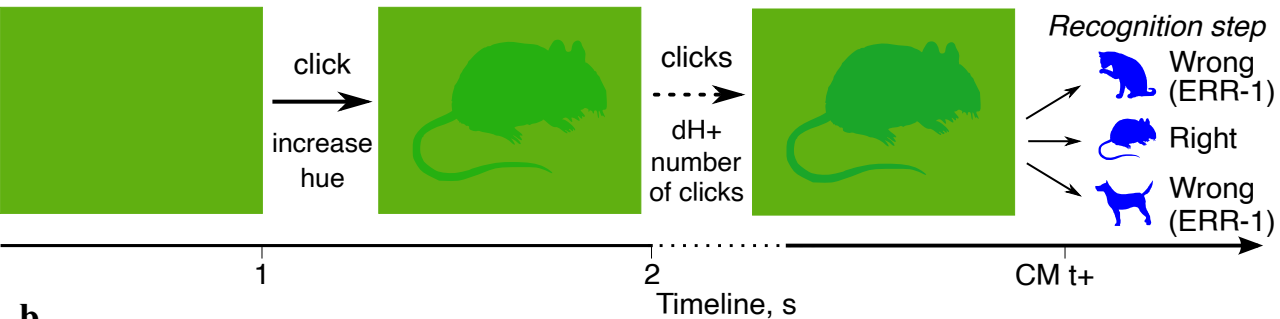**b**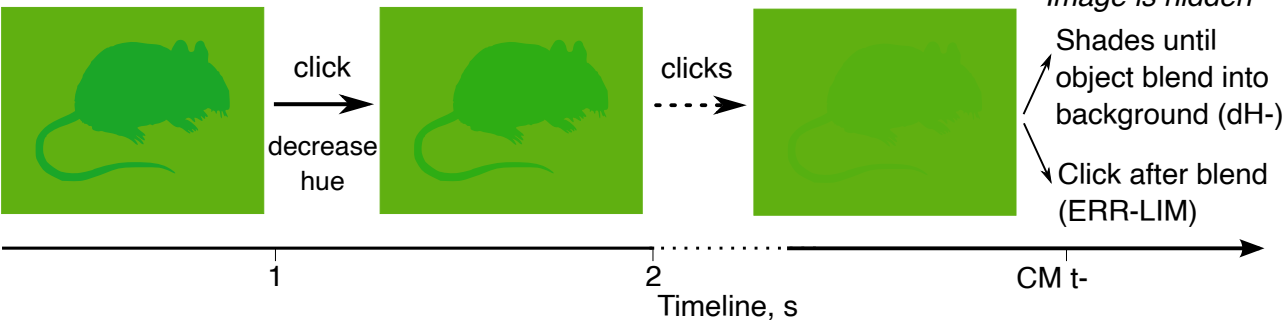

Supplement: Supplementary file 4 — Supplementary Figure S3 [file 41398_2022_2123_MOESM4_ESM.pdf]

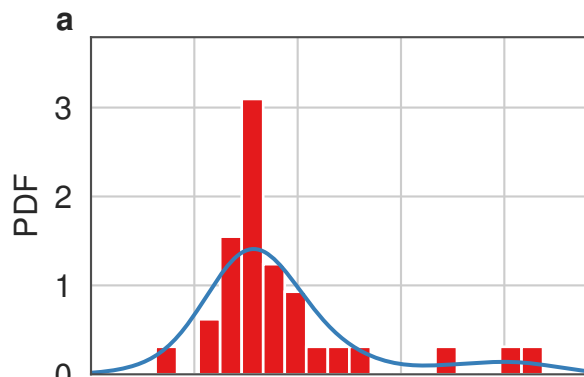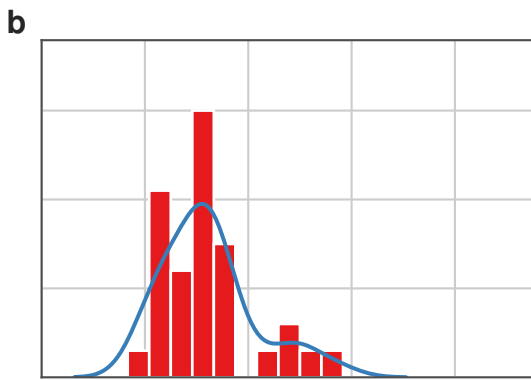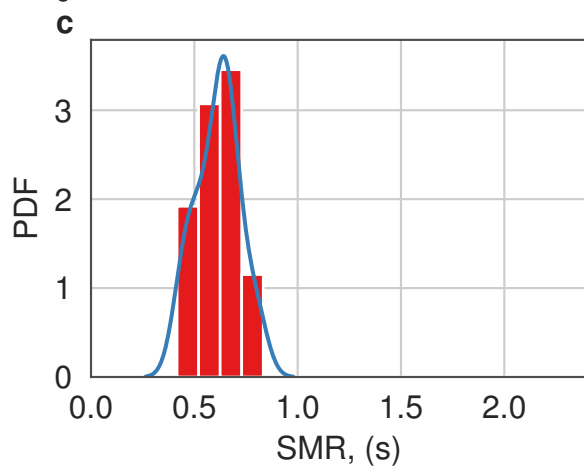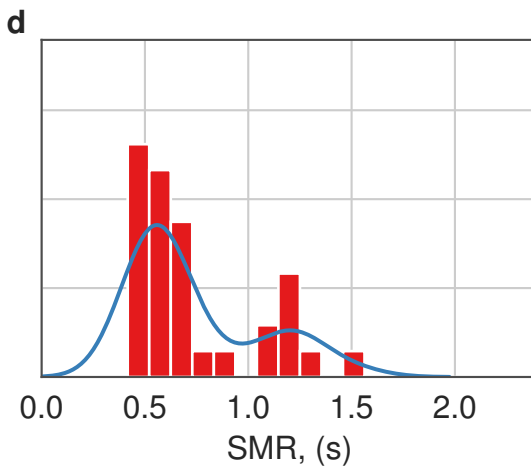

Supplement: Supplementary file 5 — Supplementary Figure S4 [file 41398_2022_2123_MOESM5_ESM.pdf]

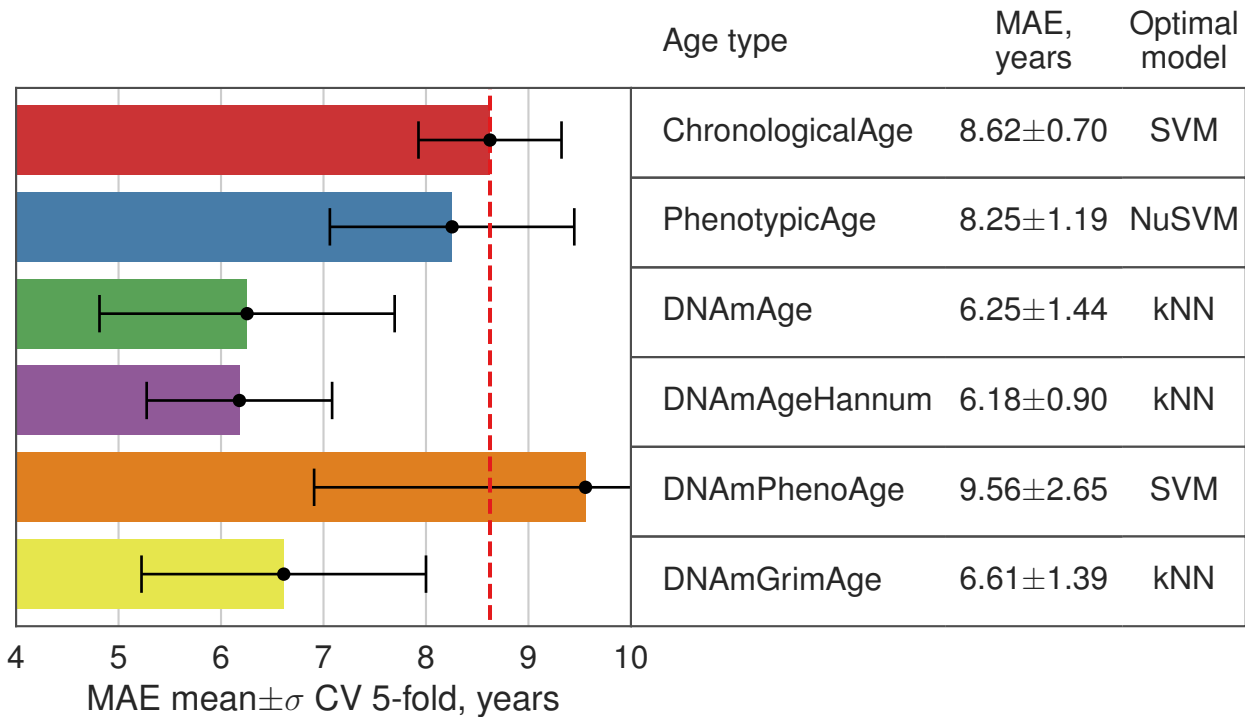

Supplement: Supplementary file 6 — Supplementary Figure S5 [file 41398_2022_2123_MOESM6_ESM.pdf]

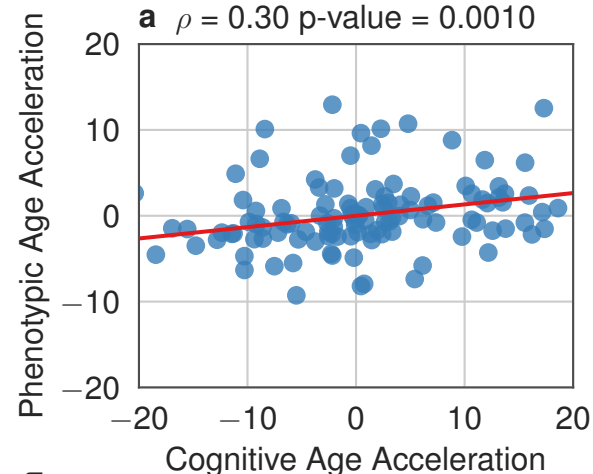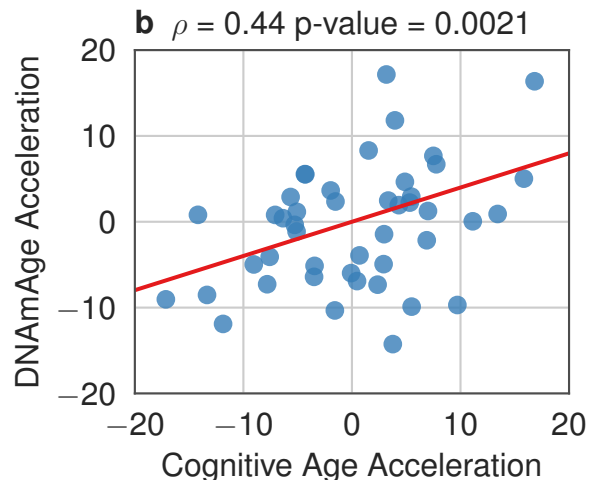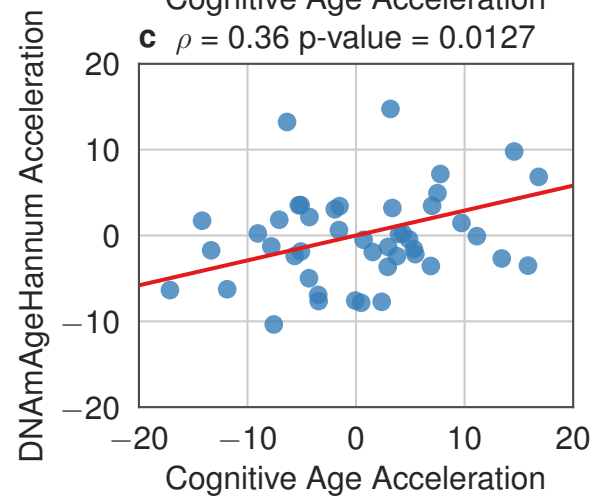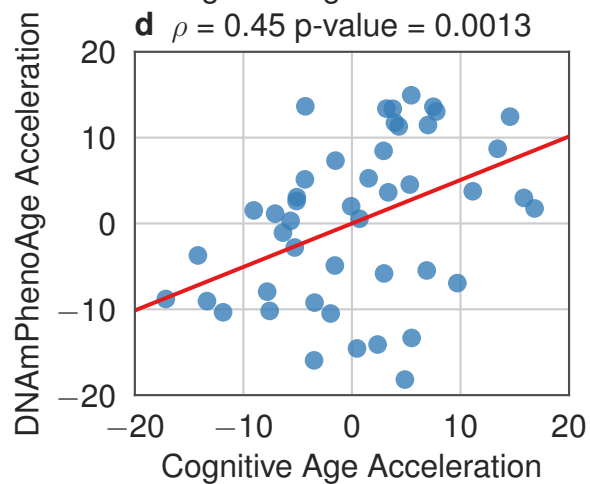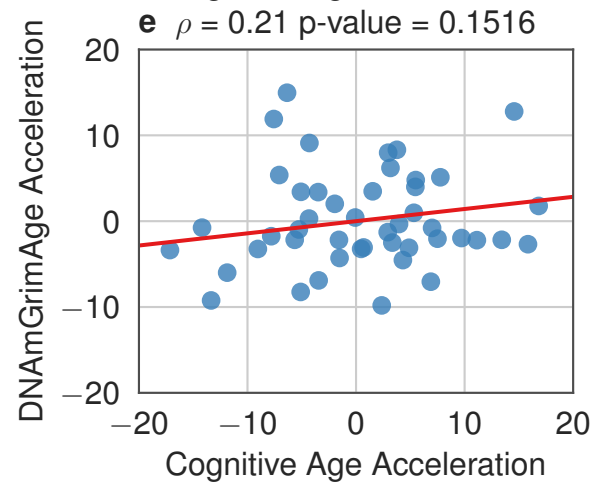

Supplement: Supplementary file 7 — Supplementary Figure S6 [file 41398_2022_2123_MOESM7_ESM.pdf]

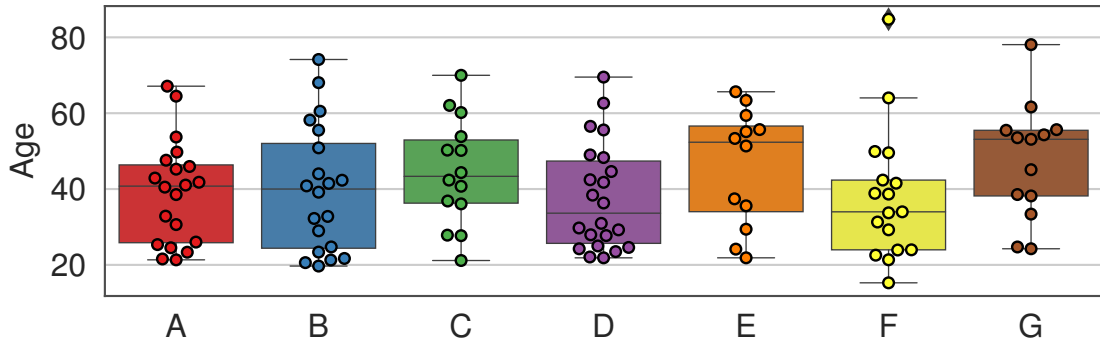

Supplement: Supplementary file 8 — Supplementary Figure S7 [file 41398_2022_2123_MOESM8_ESM.pdf]
