# Supplementary material for: A new cognitive clock matching phenotypic and epigenetic ages
Source: Transl Psychiatry. 2022 Sep 6;12:364. doi: 10.1038/s41398-022-02123-5 (PMC9444998; doi:10.1038/s41398-022-02123-5)
Supplement: Supplementary file 3 — Supplementary Figure S2 [file 41398_2022_2123_MOESM3_ESM.pdf]

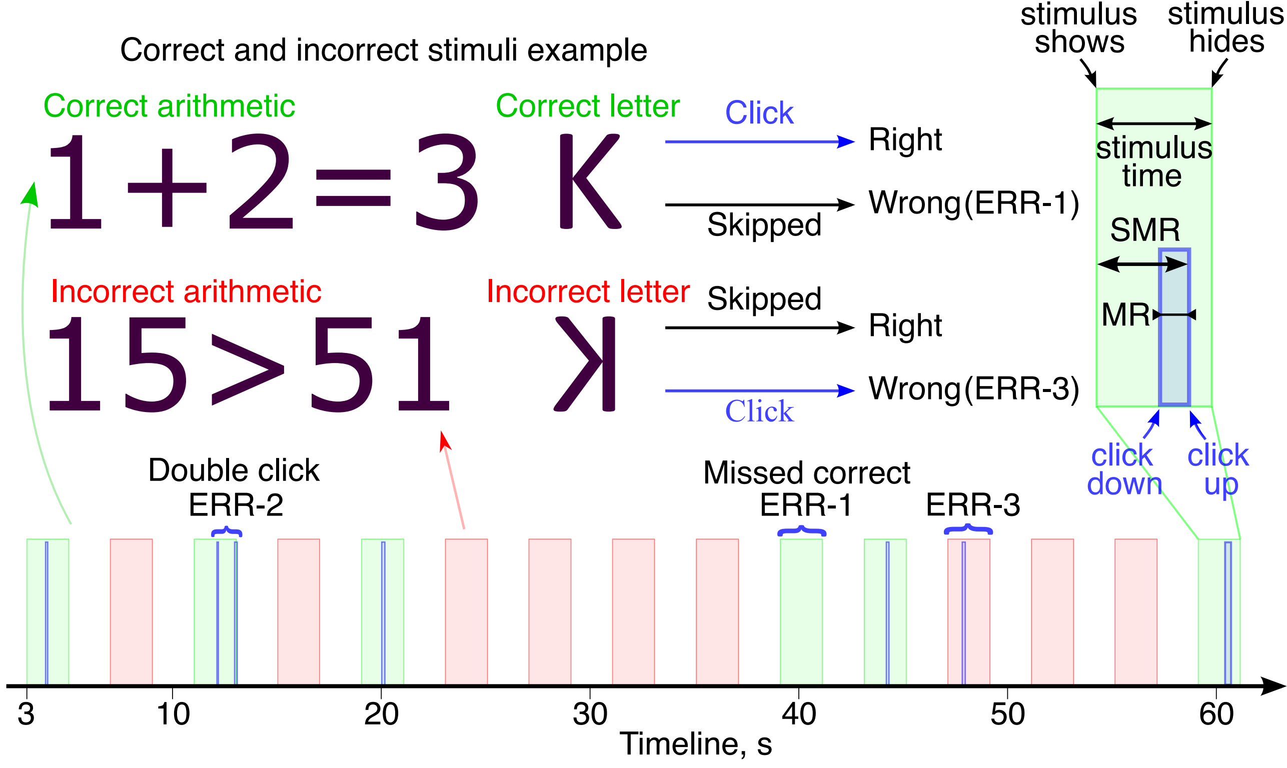
Correct and incorrect stimuli example
Correct arithmetic
1+2=3
Correct letter
K
Click
Right
Wrong(ERR-1)
Skipped
Incorrect arithmetic
15>51
Incorrect letter
Skipped
Right
Wrong(ERR-3)
Click
stimulus shows
stimulus hides
stimulus time
SMR
MR
click down
click up
Double click
ERR-2
Missed correct
ERR-1
ERR-3
3
10
20
30
40
50
60
Timeline, s
